# Editorial: Pharmacogenetic landscape in human solid cancers

**DOI:** 10.3389/fonc.2023.1343395

**Published:** 2024-01-08

**Authors:** Elena Lastraioli, Francesca Colombo, Elisa Frullanti

**Affiliations:** ^1^ Department of Experimental and Clinical Medicine, University of Florence, Florence, Italy; ^2^ Institute for Biomedical Technologies, National Research Council, Segrate, Italy; ^3^ Cancer Genomics & Systems Biology Lab, Department of Medical Biotechnologies, University of Siena, Siena, Italy; ^4^ Department of Medical Biotechnologies, Med Biotech Hub and Competence Centre, University of Siena, Siena, Italy

**Keywords:** pharmacogenetics, solid cancer, human tumours, pharmacogenomics, cancer

The response to treatment might be quite diverse within patients bearing solid tumors, mainly due to genetic factors. In order to define the key determinants of such heterogeneity and to design personalized schedules of treatment for each patient, it is of pivotal importance to study how genetic profile influences response to therapy. This is termed pharmacogenetics (or pharmacogenomics). In recent years, improvements in molecular technologies such as microarrays, genotyping and high throughput DNA sequencing gave impulse to the broader use of pharmacogenetics. These approaches hold the potential to pave the way for the development of personalized drugs to treat cancer as well as other pathological conditions (Alzheimer disease, asthma, cardiovascular diseases and many more).

This Research Topic aimed to provide an update on the effects of genetic variability on drug efficacy and toxicity, the identification and functional characterization of polymorphisms relevant to drug effects, the identification of new genetic targets for drug development and the clinical implementation of pharmacogenomics.

The effectiveness of targeted RNA sequencing in 165 tumor tissue samples was assessed by An et al. through the detection and analysis of both known and unknown oncogenic fusion genes. The oncogenic activity and therapeutic potential of these fusions were further investigated using *in vitro* assays. The findings underscored the utility of RNA panel sequencing as a theragnostic tool, providing valuable insights for identifying oncogenic fusion genes through post-sequencing analysis.

Liu et al. performed a retrospective analysis involving Chinese patients with advanced adrenocortical carcinoma (ACC) treated with mitotane for over three months. The study aimed to explore the impact of genotypic variants in *CYP2B6*, *CYP3A4* as well as *PXR* on the wide inter-individual differences observed in the mean steady-state plasma trough concentration of mitotane. The research highlighted that the cumulative dose of mitotane and polymorphisms of *CYP2B6 516* and *CYP2B6 26570* significantly affect mitotane plasma trough concentrations in Chinese ACC patients. These findings underscore the need for further prospective clinical investigations to better understand this correlation.

The review carried out by Zhao et al. systematically analyzed *RET* gene, delving into its biological aspects and elucidating its oncogenic relevance across various cancers. Additionally, recent advances in the treatment with RET kinase inhibitors and insights into the mechanisms underlying drug resistance were summarized.

Shugg et al. assessed the accuracy of the Aldy computational method to extract PGx genotypes from Whole Genome Sequencing (WGS) and Whole Exome Sequencing (WES) data for some of the major pharmacogenes. The main finding of their work pointed out that Aldy v3.3 and v4.4 called diplotypes for major pharmacogenes from clinical WES and WGS data with >99% accuracy. Therefore, the Authors propose to use the Aldy computational method in order to repurpose clinical Next Generation Sequencing data to include pharmacogenomics in the clinical management of the patients bearing solid tumors.

The group coordinated by Li et al. provided evidence that genes related to Neutrophil Extracellular Traps (NETs) were differentially expressed in healthy renal tissue compared to clear cell renal carcinoma (ccRCC) samples. Interestingly, performing an *in silico* analysis on The Cancer Genome Atlas (TCGA), International Cancer Genome Consortium (ICGC) and E-MTAB-1980 datasets the Authors showed that a 31 NET gene signature correlated with immune infiltration and drug sensitivity, suggesting it could represent a companion tool for patient stratification and management, once prospectively validated.

In brief, this Research Topic highlights information currently available concerning pharmacogenetics, the main features and their applications in oncology with a special focus on the possibility to exploit this information in the clinical setting in the near future.

## Author contributions

EL: Project administration, Supervision, Writing – original draft, Writing – review & editing. FC: Writing – original draft, Writing – review & editing. EF: Project administration, Writing – original draft, Writing – review & editing.

